# Pyridoxine-Dependent Epilepsy and Antiquitin Deficiency Resulting in Neonatal-Onset Refractory Seizures

**DOI:** 10.3390/brainsci12010065

**Published:** 2021-12-31

**Authors:** Konrad Kaminiów, Magdalena Pająk, Renata Pająk, Justyna Paprocka

**Affiliations:** 1 Students’ Scientific Society, Department of Pediatric Neurology, Faculty of Medical Sciences in Katowice, Medical University of Silesia, 40-752 Katowice, Poland; kaminiow.k@gmail.com (K.K.); magdalenapajak9626@gmail.com (M.P.); renia.pajak@gmail.com (R.P.); 2 Department of Pediatric Neurology, Faculty of Medical Sciences in Katowice, Medical University of Silesia, 40-752 Katowice, Poland

**Keywords:** pyridoxine-dependent epilepsy, seizures, inborn errors of metabolism, metabolic epilepsy, ALDH7A1

## Abstract

Pyridoxine-dependent epilepsy (PDE) is an autosomal recessive neurometabolic disorder due to a deficiency of α-aminoadipic semialdehyde dehydrogenase (mutation in *ALDH7A1* gene), more commonly known as antiquitin (ATQ). ATQ is one of the enzymes involved in lysine oxidation; thus, its deficiency leads to the accumulation of toxic metabolites in body fluids. PDE is characterized by persistent, recurrent neonatal seizures that cannot be well controlled by antiepileptic drugs but are responsive clinically and electrographically to daily pyridoxine (vitamin B6) supplementation. Although the phenotypic spectrum distinguishes between typical and atypical, pyridoxine-dependent is true for each. Diagnosis may pose a challenge mainly due to the rarity of the disorder and the fact that seizures may not occur until childhood or even late adolescence. Moreover, patients may not demonstrate an obvious clinical or electroencephalography response to the initial dose of pyridoxine. Effective treatment requires lifelong pharmacologic supplements of pyridoxine, and dietary lysine restriction and arginine enrichment should improve prognosis and avoid developmental delay and intellectual disability. The purpose of this review is to summarize briefly the latest reports on the etiology, clinical symptoms, diagnosis, and management of patients suffering from pyridoxine-dependent epilepsy.

## 1. Introduction

Pyridoxine-dependent epilepsy (PDE) is classically diagnosed in newborns with seizures responsive to pyridoxine and resistant to anti-seizure medication. It can, however, have an atypical manifestation, with later onset, in which patients do not respond to pyridoxine immediately, but their seizures can be controlled if pyridoxine therapy is continued for several months. Although epilepsy can be avoided in most patients following the aforementioned therapy, 75% of them display significant intellectual disability and developmental delay. Its incidence may vary according to different studies, from 1:20,000 in a German study to 1:276,000 in a Dutch study and 1:783,000 in a U.K.-based study) [1]. Its often significant diagnostic delay makes it a perfect candidate for newborn screening, at least in patients with epilepsy. An early diagnosis and initiation of treatment remain the most important prognostic factors for the child’s neurological development [1,2,3].

Refractory status epilepticus (RSE), which is a status epilepticus (SE) that does not respond to adequately used first- and second-line antiepileptic drugs, as well as super-refractory status epilepticus (SRSE), which can be diagnosed if the status epilepticus lasts over 24 h or when it persists after inducing coma or recurs after its ending, can both appear in PDE. These conditions usually present with generalized convulsive SE or focal SE with impaired consciousness and can be treated with general anesthetics midazolam, propofol, and barbiturates. Other forms of pharmacological therapy can also include other anesthetics such as ketamine and isoflurane, as well as immunomodulating therapy with corticosteroids, plasmaferesis, and IV immunoglobulin. Non-pharmacological therapies, including ketogenic diet, therapeutic hypothermia, neurosteroids (allopregnalone), electroconvulsive therapy, or even transcranial magnetic stimulation, might also be used with suitable outcomes. In some cases, surgical treatment with resections of various extent is necessary. Research has shown that epileptogenic zone resections in patients with refractory focal SE have made 7 in 10 patients seizure-free [4]. Surgeries are also performed in patients with very severe epilepsy, sometimes needing lobar or multilobar resections, even including spherectomy or disconnections (e.g., corpus callosotomy) [5,6]. It is also necessary to monitor the patient with continuous electroencephalography (cEEG). Other substances have also been tested on animal models (nitric oxide; with highly contradictory results, its effect depends on the epilepsy type) and on brain cells, obtained from patients (ifendopril reduces neural excitability, in animal models alleviates the behavioral manifestations) [7,8,9].

## 2. Etiology

Pyridoxine-dependent epilepsy is one of the most common inborn errors of metabolism that results in seizures. It is caused by a deficiency of α-aminoadipic semialdehyde dehydrogenase (α-AASA) in the lysine degradation pathway [10,11,12,13,14,15,16]. It manifests as a result of mutations in the antiquitin gene (*ALDH7A1*), which is a member of the aldehyde dehydrogenase (ALDH) gene family. *ALDH7A1* gene (discovered in 2006), which is located at chromosome 5q32.2, contains a transcript of 4964 base pairs and 539 amino acids divided among 18 exons [1,10,16,17]. Thus far, over 165 pathogenic variants in *ALDH7A1* have been published [18], the most frequent (30%) is the p.E399 missense mutation in exon 14 [1,10,16,17,18]. The vast majority of patients have had biallelic pathogenic variants identified in *ALDH7A1* consistent with the autosomal recessive inheritance of the disease [2,10,19,20]. When interpreting genetic testing results, it is important to note that a common synonymous variant p.Val278Val (historical nomenclature: c.750G > A, r.748_787del) results in a cryptic splice site [2,21] and that intragenic deletions are relatively common [2,18,22]. ATQ (ALDH7A1) takes part in lysine catabolism in the brain and in the liver. Pathway of lysine catabolism occurs in two ways: through saccharopin in the liver and fibroblasts (which is the predominant pathway) and the pipecolic acid (mainly in the CNS). Depending on the lysine metabolism pathway, localization in cell compartments is also different. The pathway through saccharopin occurs mainly in the mitochondria, while the pipecolic acid pathway is mainly located in peroxisomes. The convergence of the two pathways mentioned above occurs at the level of αAASA formation, which takes place in the cytosol. Lysine is regarded as a nitrogen donor allowing the formation of glutamate out of α-ketoglutarate in the central nervous system [12,13]. Pipecolic acid formed in the pipecolic acid pathway of lysine metabolism modulates the function of GABA, which is a major inhibitory neurotransmitter [2,17,18]. The enzyme α-AASA dehydrogenase (ATQ) oxidizes α-AASA to α-aminoadipic acid, and deficiency of this enzyme leads to accumulation in the body of harmful metabolites: pipecolic acid [2,23,24], α-AASA [2,25,26] and its cyclic equilibrium partner Δ1-piperideine-6-carboxylate (Δ1-P6C) [2,27], which inactivate pyridoxal phosphate (PLP)-the active form of vitamin B6. This is particularly noticeable in the central nervous system (CNS)-the concentration of pyridoxal phosphate in the cerebrospinal fluid (PMR) is significantly lower in individuals with PDE.

Pyridoxal 5′-phosphate (PLP) is an extremely important compound, being a cofactor of many enzymatic reactions, it determines their proper course. PLP is implicated in at least 140 biochemical activities occurring in the human body, corresponding to ~4% of all classified activities [1,28]. PLP acts as a coenzyme in all transamination reactions and in certain decarboxylation, deamination, and racemization reactions of amino acids [29]. Among others, it participates in metabolic pathways of transamination and decarboxylation of neurotransmitters (γ-aminobutyric acid (GABA), dopamine, serotonin), which is of great importance in the pathophysiology of seizures [2,17,18]. PLP also plays a role in glycogen phosphorylation in the liver, kidneys, CNS, modulates the activation of steroid hormones, participates in the expression of many genes [29]. The abnormal function of antiquitin resulting in elevations of the chemical α-aminoadipic semialdehyde (α-AASA) leads to reduced activity of several enzymes in the brain that regulate the transmission of signals between neurons as well as brain development [2,16]. As mentioned, the accumulated Δ1-P6C is postulated to bind the active vitamer of pyridoxine (pyridoxal 5′-phosphate) through a Knoevenagel condensation (forming P6C–PLP chemical complex), and pharmacologic doses of pyridoxine are used to overcome the secondary pyridoxal 5′-phosphate deficiency [1,2,16,17,18,19]. Studies showed a significant reduction in PLP in cerebrospinal fluid (CSF) in patients with vitamin B6-related seizures, while plasma PLP levels were not different from normal controls and from patients with non-vitamin B6 related seizures [17]. Thus depletion of PLP seems to be mainly occurring in the brain, and CSF to plasma PLP ratio might be an adjuvant marker for diagnosis and treatment monitoring [17]. Increased levels of glutamate and decreased levels of GABA due to deficient activity of PLP-dependent glutamate decarboxylase may result in a subsequent imbalance between excitatory (glutamate) and inhibitory (GABA) neurotransmitters, which could in part account for the encephalopathy and seizure characteristics of PDE [16,17,18].

Moreover, the accumulating α-AASA and related compounds were suggested to be neurotoxic organic acids, which may contribute to the poor cognitive outcome widely described in PDE [2,17].

To sum up, mutation in the antiquitin gene contributes to the dysfunction of the system through three pathways: first, accumulation of α-AASA and its heterocyclic form Δ1-P6C as a primary consequence of ATQ deficiency; second, PLP deficiency due to accumulation of toxic lysine metabolites; third, pipecolic acid accumulation as a secondary consequence of ATQ deficiency [17].

**Figure 1:**
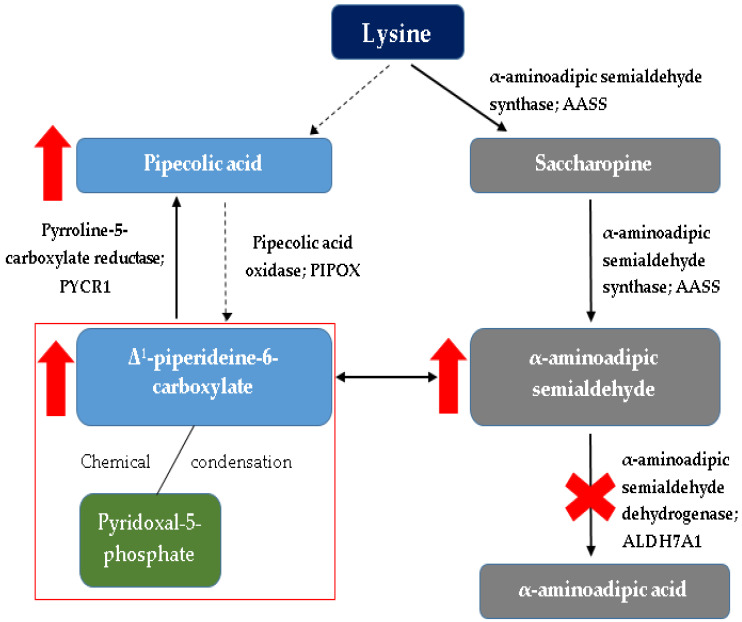
Simplified diagram of lysine metabolism with detailed (red markings) changes occurring in this cycle in pyridoxine-dependent epilepsy. The red rectangle indicates the main problem underlying the PDE, i.e., binding of the active form of vitamin B6 (pyridoxal-5-phosphate) by intermediate metabolites of lysine catabolism (Δ1-piperideine-6-carboxylate) [1,2,16,17,18,19].

A detailed diagram of lysine metabolism is shown in Figure 1.

The mutation in the *ALDH7A1* gene described above resulting in α-aminoadipic semialdehyde dehydrogenase deficiency is the most common cause of pyridoxine-dependent epilepsy, while other genetically determined causes of pyridoxine-dependent/responsive seizures to therapeutic administration of vitamin B6 can be distinguished [2,16,17,30]. For seizures that respond to vitamin B6 treatment but no biochemical or molecular markers of PDE are found, further diagnostics should be performed for the conditions broadly discussed in the differential diagnosis section, such as pyridoxal phosphate responsive epileptic encephalopathy, caused by deficiency of pyridoxamine 5′-phosphate oxidase (PNPO); tissue-nonspecific alkaline phosphatase (TNSALP) deficiency; familial hyperphosphatasia (PIGV deficiency) and hyperprolinemia type II [1,15,17,30,31,32].

Apart from these clearly defined monogenic defects, there is also a group of seizures occurring in neonates and infants that resolve after pyridoxine supplementation but are not found to be pyridoxine-dependent, which means that no recurrence of seizures after cessation of therapeutic vitamin B6 supplementation. Pyridoxine may have a non-specific therapeutic effect in patients with various types of cryptogenic and symptomatic epilepsies. In particular, patients with infantile spasms (West syndrome) or other catastrophic epileptic conditions may show a favorable response to treatment with pyridoxine in addition to conventional pharmacologic therapy [2,11,17,28,33]. What is worth emphasizing, the epileptic state is much less frequent in these patients, and usually, psychomotor and later also intellectual development of the patients remains undisturbed [2,11,17,28,33].

Some children with intractable seizures may have only partial improvement in seizure control with the addition of pyridoxine. In this situation, or in instances in which seizures recur after seizure medications are withdrawn, and pyridoxine is continued, individuals who have not had molecular confirmation of pyridoxine-dependent epilepsies should be diagnosed with “pyridoxine-responsive seizures” rather than pyridoxine-dependent epilepsy [2,3,17].

Seizures caused by hypoxic-ischemic encephalopathy (HIE), congenital central nervous system (CNS) malformations, other rare inborn errors of metabolism (IEM), genetic epilepsy syndromes, and those due to intracranial hemorrhage (ICH) or infection may not respond to vitamin B6 supplementation [2,3,11].

It is also worth noting that some individuals with pyridoxine-dependent epilepsy do not have identified mutations in the *ALDH7A1* gene. In these cases, the cause of the condition is unknown [17,34,35,36].

## 3. Symptoms

Pyridoxine-dependent epilepsy has two main types: classical and atypical. Classical PDE has its onset in the first weeks to months of life, usually manifesting with neonatal seizures, which do not respond to standard antiepileptic therapy. Some mothers also observe unusual fetal movements, starting from the second trimester of pregnancy. If untreated, PDE soon leads to recurrent status epilepticus and prolonged seizures, often with abnormal facial and eye movements. The seizures can be partial or generalized, clonic, myoclonic, or tonic, as well as with infantile spasms; sometimes, they can also be linked to febrile illness. The seizures may be preceded by periods of encephalopathy, which can also occur in older children with insufficient pyridoxine intake, related to growth or infection. EEG (electroencephalographic) activity typical for seizures may be present, even with the absence of actual seizures [1,3].

Patients may have intellectual disabilities, especially affecting expressive language. The severity of the symptoms depends mostly on the age of onset (early onset is usually associated with worse outcomes) and the diagnostic delay (late diagnosis linked to more pronounced intellectual disability). The difference is especially visible in children starting the therapy antenatally, which tend to have higher IQs than their siblings. Some cases of normal intellectual function have been reported [3,29].

Atypical PDE is distinctively harder to diagnose; the seizures usually have later onset and begin after 2 months of age. The onset might be, however, delayed, in one case until adolescence, with the diagnosis, made first in early adulthood [37]. They also initially do not respond to pyridoxine but can be controlled by it a few months later. Initial response to antiepileptic drugs, which later waves off, can be observed. In addition, after the pyridoxine therapy discontinuation, patients tend to be seizure-free for a few months. Another hallmark feature is the response to folinic acid, as some of the infants do not respond to pyridoxine in early stages, only to folinic acid. Similar to classical phenotype, the later the onset, the better the cognitive outcome [3].

## 4. Diagnosis

Pyridoxine-dependent epilepsy-*ALDH7A1* (PDE-*ALDH7A1*) should be suspected in individuals with the following clinical findings, supportive laboratory findings, and family history [3]. Table 1 shows clinical features when PDE should be suspected.

PDE is suspected in individuals with persistent seizures that cannot be controlled with antiepileptic drugs and who have no confirmed specific disorder that could be responsible for the seizures. The diagnostic and therapeutic procedure, in this case, is administering 100 mg of pyridoxine intravenously while monitoring the EEG, oxygen saturation, and vital signs [2,3,17]. In individuals with PDE, clinical seizures generally cease over a period of several minutes, and if a clinical response is not demonstrated, the dose should be repeated up to a maximum of 500 mg [2,3,17]. It is worth noting that after the administration of vitamin B6, cardio-respiratory depression may occur; therefore, it is important to monitor the patient and be ready to support him during the occurrence of adverse reactions [2,3,17]. It is suggested that sudden, severe cerebral suppression may have been the cause of the decreased level of consciousness and respiratory compromise. The mechanism of this complication of pyridoxine infusion may be related to a sudden shift in the balance of glutamate and GABA in the central nervous system. An intravenous pharmacologic dose of pyridoxine would suddenly increase the coenzyme pool, offsetting the adverse kinetics from the altered glutamate decarboxylase binding capacity. With a large amount of substrate suddenly available, an intravenous infusion of pyridoxine could lead to a sudden increase in GABA and a diffuse inhibitory state potentially ranging from excessive drowsiness to coma and electrocerebral silence [38,39].

PDE cannot be confirmed on the basis of laboratory tests, although they are necessary to exclude other causes of persistent convulsions. Patients with PDE (in addition to elevated concentrations of α-AASA and Δ1-P6C) may present with lactate acidosis, hypoglycemia, electrolyte disturbances, or hypothyreosis. Often abnormalities in the distribution of neurotransmitters in the CNS and abnormal amino acid concentrations (serum and PMR) are found [1,2,3,17,40]. Increased glutamine concentrations and decreased GABA and 5-methyltetrahydrofolate (5-MTHF) concentrations in CSF can also be observed. Importantly, most of these abnormalities compensate during vitamin B6 administration, so do not delay in securing material for laboratory testing (e.g., serum or cerebrospinal fluid) [1,2,3,17,40].

Unfortunately, the electroencephalographic recording is variable and non-specific. There is not a well-defined electrographic signature that is pathognomonic for either neonatal-onset or late-onset PDE-*ALDH7A1*, which emphasizes the importance of testing for this disease in the absence of an established etiology [2,41]. It can be completely normal, high voltage delta waves, burst-suppression activity (SBA) may occur, and hypsarrhythmia is rare. As with changes in laboratory findings, the EEG usually normalizes after treatment with pyridoxine [1,29]. It is possible to obtain an immediate improvement in the EEG with intravenous pyridoxine administration. However, this is not a criterion for the diagnosis of PDE, as there are both cases of patients with PDE who had a normal EEG and those patients in whom, despite the presence of changes in the EEG (i.e., burst-suppression activity) and intravenous pyridoxine was administered without any improvement in EEG or resolution of seizures [11,41].

Biochemical markers are the most widely used in the diagnosis of pyridoxine-dependent seizures. Both α-AASA, as well as Δ1-P6C, are considered biochemical markers of PDE. Currently, α-AASA and Δ1-P6C are considered reliable PDE biomarkers that should be determined in every case of persistent seizures in infants and young children [1,15,17]. However, it should be remembered that diagnostics should not delay the start of treatment with vitamin B6 [1,15,17]. Their concentration can be determined in serum, urine, and CSF. The concentration of α-AASA depends on the age of the patient, the type of ALDH7A1 gene mutation, and the amount of lysine consumed with food. These substances are thermally unstable. They decompose at room temperature within several hours; therefore, the collected material needs to be stored in a refrigerator, and ideally, it should be frozen immediately after collection. In addition to α-AASA and Δ1-P6C, pipecolic acid (PA) is a non-specific marker of ALDH7A1 deficiency, as increased levels are also found in other metabolic diseases. These include: peroxisome dysfunction, hyperlysinemia, hyperprolinemia, and it also occurs in liver dysfunction. Some patients who are tested after pyridoxine treatment may have normal levels of PA [19,42]. New biomarkers have been reported, including 6-oxo-pipecolate, although its role in diagnosis or treatment monitoring has yet to be established [43,44].

In many patients, MRI findings include hypoplasia of corpus callosum, mega cisterna magna, intracerebral hemorrhages, ventriculomegaly, cerebellar hypoplasia, incomplete/delayed myelination, white matter lesions, some cases of hydrocephalus, subependymal cysts, and hypoplasia of optical chiasm have also been recorded. A normal MRI image, however, does not exclude PDE [37,45,46].

Molecular genetic testing is the only reliable method for carrier screening of family members, prenatal diagnosis, and, importantly, confirming the clinical diagnosis of PDE [1,2,15,17,47,48]. Biochemical testing should be performed when a single pathogenic variant or a variant of uncertain significance is identified [2]. Prospective genetic evaluations of patients with epilepsy have identified patients with PDE-*ALDH7A1* that were otherwise not diagnosed [1,2,15,47,49]. Recent recommendations have included testing all children with seizures of unknown etiology [1,2,15,47,49].

## 5. Differential Diagnosis

Pyridoxine-dependent epilepsy (PDE-*ALDH7A1*) should be considered as a cause of intractable seizures occurring in all ages for which an underlying lesion has not been defined. Clinicians can suspect this pathology in particular when there is no convincing evidence of hypoxic-ischemic encephalopathy or other identifiable underlying metabolic disturbance in neonates with seizures and encephalopathy [49].

The differential diagnosis of PDE-*ALDH7A1* includes:

- PLP-responsive epileptic encephalopathy due to PNPO deficiency;
- Neonatal/infantile hypophosphatasia (TNSALP deficiency);
- Familial hyperphosphatasia (PIGV deficiency), although only a few patients have been reported presenting a response to pyridoxine or PLP [50] as well as nutritional vitamin B6 deficiency and yet unidentified conditions [17].

Pyridoxamine-5′-phosphate oxidase (PNPO) deficiency is an autosomal recessive epileptic encephalopathy responsive to pyridoxal 5′-phosphate (PLP). Dysfunctional variants of *PNPO* are unable to catalyze the production of PLP; therefore, PLP must be supplemented in doses of 30 mg/kg/day [51]. This treatment provides seizure control in all examined cases, although approximately half of patients affected with PLP-dependent epilepsy suffer from an intellectual disability or delayed development. Main symptoms of PNPO include: refractory seizures in the first year of life, pre-maturity, and fetal distress [51].

Pyridoxine-dependent epilepsy-*PLPBP* (PDE-*PLPBP*) or PLP binding protein deficiency is caused by a recessive mutation resulting in pathogenic variants of *PLPBP.* Inefficacy and deficiency of this enzyme are manifested by encephalopathy with early-onset intractable seizures responsive to pyridoxine and/or PLP. Additional clinical features include delayed development and structural brain abnormalities, most notably cyst-like structures adjacent to the anterior horns and simplified gyral pattern. Biochemical tests revealed hyperlactatemia and hyperglycinaemia in some cases [52].

Hypophosphatasia (HPP) is caused by mutations in genes encoding tissue-nonspecific alkaline phosphatase (*TNSALP*). TNSALP is expressed in the liver, kidney, teeth, and bone. Its substrates include inorganic pyrophosphate, phosphoethanolamine (PEA), and pyridoxal-5’-phosphate (PLP)/vitamin B6. TNSALP plays an important role in mineralization as well as in the growth and development of bones and teeth. Therefore, HPP can result in hypo-mineralization leading to limbs deformation, even in some cases to near-absence of bones and skull in patient’s body, respiratory insufficiency, and seizures due to vitamin B6 deficiency. Other symptoms include: hypercalcemic crisis, ricket, craniosynostosis, odontohypophosphatasia, failure to thrive, and growth retardation. Fractures are common and manifest themselves with unexplained fatigue and chronic pain. Major hallmarks of HPP are high levels of calcium, low alkaline phosphatase (ALP) level as well as elevated PLP and PEA levels. Treatment of this disease is based on the administration of a subcutaneous bone-targeted recombinant form of TNSALP (asfotase alfa), which is specifically targeted to mineralized tissues [53].

A comprehensive summary of the disorders that should be considered in the differential diagnosis of pyridoxine-dependent epilepsy is presented in Table 2.

## 6. Management

Effective management of PDE requires lifelong pharmacologic supplementation of pyridoxine. This treatment provides seizure control; however, at least 75% of individuals have developmental delay and intellectual disability in spite of vitamin B6 administration. Therefore the therapy has been extended with other specifics [13].

Pyridoxine supplementation remains a first-line treatment for PDE. Due to the rarity of the disorder, it is impossible to conduct controlled studies, which are crucial for the evaluation of the optimal dose [49]. The newest guidelines recommend the following doses:

- Newborns—100 mg/day;
- Infants—30 mg/kg/day (maximum dose-300 mg/day);
- Adults—200–500 mg/day [2].

Infants with acute seizures should be provided with a single (or more) 100 mg dose of pyridoxine given intravenously [15]. This particular treatment is associated with an increased risk of apnea; therefore, it should be administrated in a controlled environment with the readiness of respiratory support and, if available, access to EEG [17]. Some patients with PDE can experience breakthrough seizures during febrile illnesses. During the first three days of such illness, the dose can be doubled [2].

Pyridoxine in excessive doses can damage the peripheral nervous system, which leads to reversible sensory neuropathy [49,54]. Therefore all patients treated with supplementation of this vitamin should have clinical screening for neuropathy. It includes electrodiagnostic testing, which should be conducted based on clinical suspect of neuropathy or every 1–2 years for patients on a high dose of pyridoxine (>500 mg/day) [2].

Prenatal supplementation of pyridoxine in the fetus at risk (when diagnostic was not pursued during pregnancy) or confirmed with PDE was shown to be effective in preventing seizures, and in some cases (missense mutation in gene E399Q), it resulted in suitable further development of patients [55]. The guidelines recommend 100 mg/dose for pregnant women [2], which is also used in the treatment of hyperemesis gravidarium. The therapy should begin early during pregnancy. Genetic tests should be performed in 11 or 12 gestational age, and if mutation of ATQ has been ruled out, the treatment should be stopped. Side effects for the fetus in this management have not been reported [17].

If pyridoxine has failed to interrupt the initial acute status epilepticus in a patient suspected with pyridoxine responsive seizure, pyridoxal phosphate should be administered. Lack of response for pyridoxine is usually associated with PNPO (pyridoxal phosphate-responsive pyridoxine phosphate oxidase) deficiency, although the idiopathic reaction can also occur [56,57,58]. Recommended dose: 30 mg/kg/day [2]. It should be noted that a high dose of PLP may cause liver dysfunction and convulsions [15].

In the presence of incomplete pyridoxine responsiveness or breakthrough seizures, folic acid should be administered. The underlying mechanism of this therapy is unknown. A high dose of folic acid may cause acerbation of seizures [17]. Recommended dose: 3–5 mg/kg/day [2].

Antiquin (AQT) deficiency results in the accumulation of intermediates arising from lysine inadequate degradation such as α-aminoadipic semialdehyde (α-AASA), pipecolic acid, and Δ^1^-piperideine-6-carboxylate (P6C). Although pyridoxine supplementation is in most cases effective to prevent seizures, lysine degradation intermediates remain elevated throughout the therapy [14]. It is suggested that those metabolites can lead to developing impairments in patients treated for PDE. Unlike treatment with pyridoxine, a lysine-restricted diet was shown to be effective in decreasing the level of lysine-derived substrates and can contribute to the improvement of cerebral functions [15]. However, neurological outcomes associated with a lysine-restricted diet were reported only in 10 cases out of the 27-patient group [2]. Therefore this treatment can only be add-on therapy for patients supplementing pyridoxine. It is important to note that plasma lysine levels should be in a low normal age-dependent reference range [2].

In order to control daily protein intake, lysine-free amino acid formulas are recommended for patients with PDE. Many commercially available formulas are registered for patients with glutaric aciduria type I (GA-1). This disease is caused by insufficiency or absence of functional glutaryl-CoA dehydrogenase (GCDH), resulting in the accumulation of tryptophan degradation byproducts [11]. Therefore, those specifics contain a low amount of tryptophan. Without additional supplementation, symptoms of this amino acid deficiency can display. A natural protein-restricted diet can be considered if formulas are unavailable or the patient does not tolerate it [17].

Arginine competes with lysine for cationic amino acid transporter 1 (CAT1), which is present in the blood-brain barrier, and for mitochondrial ornithine carriers (ORNT1, ORNT2) present in mitochondrial membranes. Therefore, it has been suggested that pharmacological doses of arginine could reduce excess lysine influx through the blood-brain barrier, resulting in a decreased level of lysine intermediates in CSF [15]. Research has shown that supplementation of arginine and pyridoxine, even without a lysine-restricted diet, has led to the general improvement of patients’ state, as well as motoric and verbal functions [59]. The guidelines recommend 200 mg/day as an initial dose of arginine provided alone or in combination with a lysine-restricted diet [2].

Table 3 provides a brief summary of the management of PDE (ATQ deficiency).

## 7. Conclusions

Due to significant phenotypic heterogeneity between patients, as well as limited clinician awareness of PDE deficiency (and especially ATQ) as a potentially treatable but rare cause of epilepsy, diagnosis can be challenging. Considering the rarity of the condition, the number of reliable studies conducted on a representative sample group is small, and consequently, the available evidence is limited. Since prospective randomized controlled trials (RCTs) in rare diseases are difficult, it is important that researchers and clinicians from around the world share their insights into the symptoms, diagnosis, and treatment regimens of PDE. Multicenter studies would undoubtedly be important because they provide the treatment modality and the results achieved. A regimen could be developed to facilitate clinical decision making and improve the care for patients with PDE-*ALDH7A1* in a standardized manner. The more we know, the easier it will be to diagnose patients and incorporate adequate treatment that will limit further damage, improve the prognosis and quality of life of patients, mainly by maintaining optimal neurological outcomes.

## Figures and Tables

**Table 1:** Clinical features when PDE should be suspected [3].

1. Seizures in any child younger than age one year without an apparent brain malformation or acquired brain injury as the cause of the epilepsy
2. Cryptogenic seizures in a previously normal infant without an abnormal gestational or perinatal history
3. Neonates with a phenotype suggestive of hypoxic-ischemic encephalopathy and with difficult-to-control seizures
4. The occurrence of long-lasting focal or unilateral seizures, resistant to anti-seizure medications, often with partial preservation of consciousness
5. Infants and children with seizures that are partially responsive to anti-seizure medications, in particular, if associated with developmental delay and intellectual disability
6. Signs of encephalopathy include irritability, restlessness, abnormal crying, and vomiting preceding and/or following the actual seizures
7. Infants and children with a history of seizures responsive to folinic acid
8. Individuals with a history of transient or unclear response of seizures to pyridoxine

**Table 2:** Conditions causing vitamin B6-dependent/responsive epilepsies [1,15,17,30,31,32,50,51,52,53].

Condition/Gene Defect	PDE (ATQ Deficiency)	PLP-Responsive Epileptic Encephalopathy (PNPO Deficiency)	Hypophosphat-Asia (TNSALP Deficiency)	Familial Hyperphosphatasia (PIGV Deficiency)	Hyperproline-mia type 2 (P5CD Deficiency)
**Gene (location)**	*ALDH7A1* (5q23.2)	*PNPO* (17q.21.32)	*ALPL* (1p36.1–34)	*PVIG* (1p.36.11), *PIGO* (9p13.3), *PGAP2* (11p15.4)	*P5CDH* (1p36.13)
**Clinical presentation**	Neonatal/infantile epileptic encephalopathy	Neonatal epileptic encephalopathy	Osteomalacia, hypercalcemia, hypo-phosphatemia, in severe forms also neonatal epileptic encephalopathy	DD/ID, seizures, dysmorphic facial feature, brachytelephal-angy	Developmental delay, intellectual disability, seizures, mild ataxia
**Diagnostic/biomarkers**	U-AASA, P-Pip P-AASA, P-P6C	Urinevanillyl-lactate; CSF HVA, HIAA, Threonine, Glycine	P-ALP low, P-PLP high, U-Phosphatidyl-ethanolamine high	P-ALP high	P-Proline, U-P5C
**Treatment**	Pyridoxine	Pyridoxal phosphate	Pyridoxine	Pyridoxine	Pyridoxine
**Outcome on treatment**	(Near) complete seizure control, DD/ID	Improvement of seizures, severe DD	Seizure control, (lethal) bone disease	Seizure control	Non-progressive DD/ID, occasional seizures

ALP = alkaline phosphatase; ATQ = antiquitin; CSF = cerebrospinal fluid; DD/ID = developmental delay/intellectual disability; HIAA = hydroxyindole acetic acid; HVA = homovanillic acid; P-AASA = plasma α-aminoadipic semialdehyde; PIGV = phosphatidylinositol glycan anchor biosynthesis type V; PLP = pyridoxal phosphate; PNPO = pyridox(am)ine-phosphate oxidase; P-Pip = plasma pipecolic acid; P5C = pyrroline 5-carboxylate; P5CD = Δ1-pyrroline 5-carboxylate dehydrogenase; P-P6C = plasma Δ1-piperideine-6-carboxylate; TNSALP = tissue-nonspecific alkaline phosphatase; U-AASA = urinary α-aminoadipic semialdehyde.

**Table 3:** Medications used in PDE treatment [2,17].

Medication	Route of Administration	Dosage	Indication	Monitoring	Side Effects
Pyridoxine	i.v.	100 mg single dosage	Interruption of initial status epilepticus, or of prolonged breakthrough seizures	EEG if available	May result in respiratory arrest. Administer upon availability of respiratory support
Pyridoxine	Oral/enteral	15–30 mg/kg/day Div in up to 3 single doses Up to 300 mg/day in neonates and 500 mg/day in adults	Long-term treatment	Clinical and electrophysiological signs of neuropathy	Continue with dosages above the range only if high dosage has proven essential for effective seizure control
Pyridoxine	Prenatal maternal	100 mg/day	Prevention of intrauterine seizures and irreversible brain damage. Start in early pregnancy, continue throughout pregnancy in case of positive prenatal diagnosis or if no prenatal diagnosis has been performed	Monitor for seizures and encephalopathy after delivery in NICU/SCN setting. Consider IV pyridoxine in case of neonatal seizures	Continue oral/enteral pyridoxine supplementation at 30 mg/kg/day immediately after birth and immediately initiate biochemical and molecular genetic investigations to prove or rule out ATQ deficiency
Pyridoxal phosphate	Oral/enteral	30 mg/kg/day divided in up to 3 single dosages	Interruption of initial status epilepticus: additional to IV pyridoxine in case pyridoxine initially failed to control seizures. Long-term treatment: Alternative to pyridoxine	Same as pyridoxine (EEG if available)	Same as pyridoxine
Folinic acid	Oral/enteral	3–5 mg/kg/day divided in up to 3 SD	Additional therapy if pyridoxine or PLP failed to control seizures	No particular monitoring	None

Abbreviations: i.v. = intravenous administration; div = divided; NICU = Neonatal Intensive Care Unit; SCN = Special Care Nursery; SD = single dosage.
